# Traditional Chinese medicine decreases the obstructive uropathy risk in uterovaginal prolapse: A nationwide population-based study

**DOI:** 10.1097/MD.0000000000012369

**Published:** 2018-09-21

**Authors:** Yin-Jen Chang, Wen-Chi Chen, Jen-Huai Chiang, Yuan-Chih Su, Kao-Sung Tsai, Kee-Ming Man, Ming-Yen Tsai, Yung-Hsiang Chen, Huey-Yi Chen

**Affiliations:** a Departments of Chinese Medicine, Urology, Medical Research, Obstetrics and Gynecology, and Anesthesiology; b Graduate Institute of Integrated Medicine, College of Chinese Medicine, Research Center for Chinese Medicine & Acupuncture; c Management Office for Health Data, China Medical University Hospital; d College of Medicine, China Medical University; e Department of Applied Cosmetology, Hungkuang University; f Department of Chinese Medicine, Kaohsiung Chang Gung Memorial Hospital and Chang Gung University College of Medicine, Kaohsiung; g Department of Psychology, College of Medical and Health Science, Asia University, Taichung, Taiwan.

**Keywords:** nationwide population-based study, obstructive uropathy, traditional Chinese medicine, uterovaginal prolapse

## Abstract

Traditional Chinese medicine (TCM) is a popular treatment for voiding dysfunction in Eastern countries. However, no previous studies have investigated the effects of TCM on preventing obstructive uropathy in uterovaginal prolapse women. We conducted a large-scale nationwide population-based cohort study to investigate the relationship between TCM and obstructive uropathy in uterovaginal prolapse women. This is a retrospective cohort study with the Taiwan National Health Insurance Research Database (NHIRD). The study population was newly diagnosed uterovaginal prolapse patients between 1997 and 2010 year. Among patients, 762 uterovaginal prolapse patients in this cohort. Significant adjusted HRs of urine retention or hydronephrosis in Cox proportional hazard models were uterovaginal prolapse (hazard ratio [HR]: 1.74, 95% confidence intervals [CI]: 1.43–2.14), age 40 to 64 years (1.51, 1.01–2.27), ≥60 years (3.52, 2.32–5.34), DM (1.52, 1.23–1.89), hypertension (1.38, 1.13–1.7), constipation (1.35, 1.05–1.75), urinary tract calculi (1.54, 1.06–2.23), and TCM users (0.34, 0.28–0.41). The Kaplan–Meier analysis showed a higher incidence rate of urine retention or hydronephrosis in the uterovaginal prolapse cohort compared with that of the without uterovaginal prolapse cohort. The results of this nationwide population-based study support a relationship between TCM and a reduced risk of obstructive uropathy in uterovaginal prolapse women.

## 1 Introduction

Pelvic organ prolapse (POP), the herniation of the pelvic organs to or beyond the vaginal walls,^[1,2]^ is a common condition in older women. According to previous studies, 32% to 98% of middle-aged and older women are reported to have some degree of pelvic organ prolapse on examination.^[3–5]^ Uterovaginal prolapse, also called apical compartment prolapse, means the descent of the apex of the vagina into the lower vagina, to the hymen, or beyond the vaginal introitus.^[6]^

Advanced prolapse may cause anatomic distortion of lower urinary tract including urethral kinking, resulted in impaired urine flow and an elevated postvoid residual.^[7]^ According to previous studies, urinary splinting was reported by 5% to 12% of women with stage II anterior prolapse and 23% to 36% of those with stage III or IV anterior prolapse.^[8]^ This anatomic obstruction leads to dysfunctional voiding, which would increase the obstructive uropathy, including clinical conditions of urinary retention^[9]^ or hydronephrosis.^[10–13]^ The treatment for uterovaginal prolapse women to prevent obstructive uropathy include alpha-adrenergic blocker medications, vaginal pessary, and pelvic reconstructive surgery. α-adrenergic blocker such as terazosin, tamsulosin might act on the urethra alpha_1_-adrenergic receptor, thus reducing bladder outlet obstruction and post-void residual (PVR) urine. About 67% to 84% patients reported that the treatment was beneficial.^[14–16]^ Vaginal pessaries are silicone devices in a variety of shapes and sizes, which support the pelvic organs. The use of pessary was associated with relief of urinary retention in 75% patients.^[17,18]^ Surgical repair of uterovaginal prolapse is depended on patient's condition. There are many different surgeries for uterovaginal prolapse, such as abdominal sacral colpopexy, laparoscopic sacrocolpopexy, vaginal surgical approach with synthetic mesh, and transvaginal apical repair procedures. An elevated preoperative PVR normalizes after surgical correction of prolapse in over 90% of women.^[19]^ However, alpha-blocker has some side effects including dizziness and rhinitis, pessaries must be removed and cleaned on a regular basis and local infection is one of the contraindications, surgical treatment incurs the risk of complications and recurrence,^[20]^ which reduce the willingness of patients to receive treatment.

Traditional Chinese medicine (TCM) is a popular treatment for voiding dysfunction in Eastern countries, including Taiwan.^[21–23]^ For example, Bu-zhong-yi-qi-tang (BT) is a classical formula for the treatment of spleen-qi descending, visceroptosis with hyposplenic qi, and uterovaginal prolapse in TCM and has been identified as an effective drug for the treatment of TCM spleen-qi deficiency in clinical practice. The restorative effects of BT were observed in certain metabolic pathways, such as the energy, protein, and glycolytic metabolisms.^[24]^ In Taiwan, >62% of urolithiasis patients use TCM.^[25]^ Previous studies have reported that several Chinese herbs can be used to treat urolithiasis.^[26]^ Some formulae have been proved that can inhibit the severity of calcium oxalate crystallization,^[27]^ some can suppress the growth of crystals, and reduce the incidence of stones in animal models.^[28,29]^ Furthermore, some formulae demonstrate a nephroprotective effect.^[30,31]^ However, no previous studies have investigated the effects of TCM on preventing obstructive uropathy in uterovaginal prolapse women. Therefore, we conducted a large-scale study, using nationwide population-based cohort study, to investigate the relationship between TCM and obstructive uropathy in uterovaginal prolapse women.

## 2 Materials and methods

### 2.1 Data sources and study subjects

This is a retrospective cohort study. We used the Taiwan National Health Insurance Research Database (NHIRD) to analysis data. Taiwan launched a compulsory, social insurance program, the NHI program, to provide health care for >99% of the 23.75 million residents in 1995. The longitudinal Health Insurance Database 2000 (LHID2000), which was used in this study, comprises the medical information of 1 million insurant, randomly sampled from the registry of all beneficiaries for the year 2000. The claims data in the LHID2000 was extended to December 31, 2011, and retrospectively collected until January 1, 1996. There were no significant differences in the distributions of sex and age between the original claims data and the sampled data. The diagnosis codes in the NHIRD were in accordance with the International Classification of Diseases, 9th Revision, Clinical Modification (ICD-9-CM). This study was approved to fulfill the condition for exemption by the Institutional Review Board (IRB) of China Medical University (CMUH-104-REC2–115). The IRB also specifically waived the consent requirement.

The study population was newly diagnosed uterovaginal prolapse patients between 1997 and 2010 year. Population with uterovaginal prolapse should have at least 2 ambulatory or in-patient's claims with diagnosis of ICD-9-CM code 618.2 or 618.3 or 618.4. Among patients in the LHID, 762 uterovaginal prolapse patients in this cohort. We used 1:4 frequency match by age and index year to selected random without diagnosis of uterovaginal prolapse as compared cohort group (n = 3048). The index date defined the first diagnosis date of uterovaginal prolapse between 1997 and 2010 year for cohort group; random give the date between 1997 and 2010 years as index date for compared cohort. All subjects were follow-up from the entry date until first diagnosis date of urine retention or hydronephrosis, withdrawal from the database, or December 31, 2011. We excluded who had accepted pelvic reconstructive surgery before the index date.

### 2.2 Outcome and relevant variables

The primary outcome was the event of obstructive uropathy, including clinical conditions of urine retention (ICD-9-CM 788.20) or hydronephrosis (ICD-9-CM 591) during the follow-up period (1997–2011). Relevant variables were age, drug used, TCM used and comorbidities, including diabetes mellitus (ICD-9-CM: 250), hypertension (ICD-9-CM: 401), cerebrovascular accident (ICD-9-CM: 434.91, 434.11), asthma (ICD-9-CM: 493), constipation (ICD-9-CM: 564.0), and urinary tract calculi (ICD-9-CM: 592.0, 592.1, 592.9, and 594.1). All comorbidities were defined before the index date and should have at least 2 ambulatory or in-patient's claims.

### 2.3 Exposure to TCM

The NHIRD records detailed prescription information for both TCM and western medicine codes. Patients using TCM after initial diagnosis of uterovaginal prolapse disease were defined as TCM users, whereas those no treated were considered as TCM non-users.

### 2.4 Statistical analysis

Difference in demographic characteristics, treatment, TCM used, and comorbidities between the cohort and compared cohort and the reference cohort were tested by chi-square test for categorical variables and *t* test for continuous variables. We estimated hazard ratio (HR) and 95% confidence intervals (95% CI) by Cox proportional hazard model. The Kaplan–Meier method was used to estimate the obstructive uropathy proportion for the cohort and compared cohort group. All analyses were performed using SAS statistical software (version 9.4; SAS Institute, Inc., Cary, NC), and the results were considered statistically significant when two-tailed *P* values were <.05.

## 3 Results

Baseline sociodemographic factors, comorbidities and treatment between cohort and compared cohort group are shown in Table 1. Uterovaginal Prolapse cohort was linked with the following factors: higher proportions of constipation; urinary tract calculi; TCM used; and alpha-blocker used. The means of age were 55.15 (13.21) and 55.07 (13.28) for uterovaginal prolapse cohort group and compared cohort group, respectively. Table 2 displays uni- and multivariable Cox proportional hazard models in a cohort of uterovaginal prolapse versus non-uterovaginal prolapse population during 1997 to 2011. Significant adjusted HRs of urine retention or hydronephrosis in Cox proportional hazard models were uterovaginal prolapse (HR: 1.74, 95% CI: 1.43–2.14), age 40–64 years (1.51, 1.01–2.27), >60 years (3.52, 2.32–5.34), DM (1.52, 1.23–1.89), hypertension (1.38, 1.13–1.7), constipation (1.35, 1.05–1.75), urinary tract calculi (1.54, 1.06–2.23), and TCM users (0.34, 0.28–0.41).

**Table 1:**
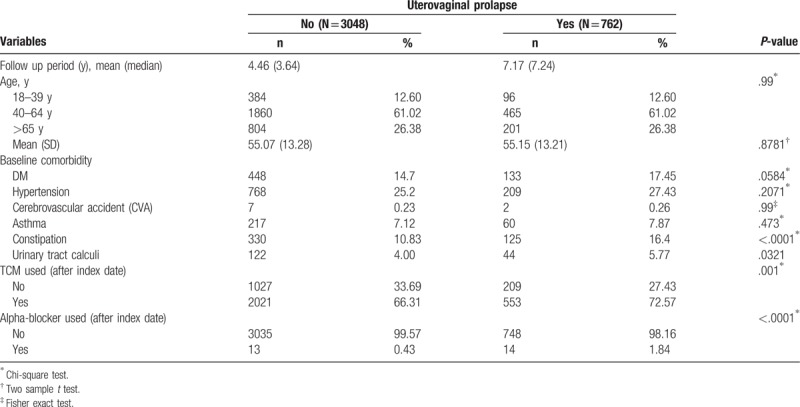
Demographic characteristics and comorbidity in patients with and without uterovaginal prolapse.

**Table 2:**
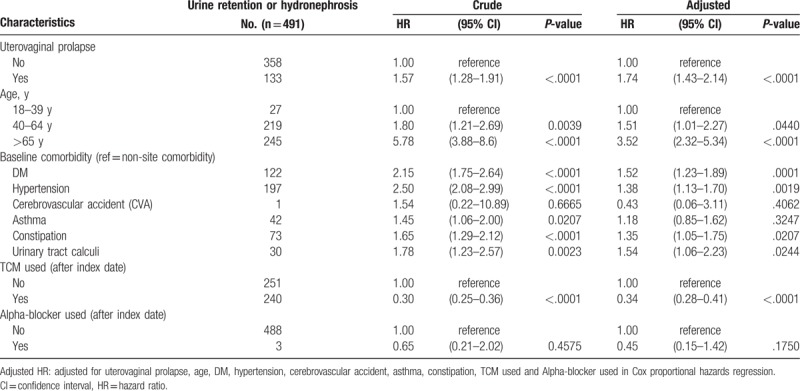
Cox model measured hazard ratio and 95% confidence intervals of urine retention or hydronephrosis associated with uterovaginal prolapse.

Table 3 shows stratified by with and without uterovaginal prolapse, uterovaginal prolapse group using TCM had lower risk of urine retention or hydronephrosis than non-TCM users (aHR:0.2, 95% CI: 0.14–0.29). And non-uterovaginal prolapse group also had lower risk of urine retention or hydronephrosis than non-TCM users (aHR: 0.41, 95% CI: 0.33–0.5).

**Table 3:**
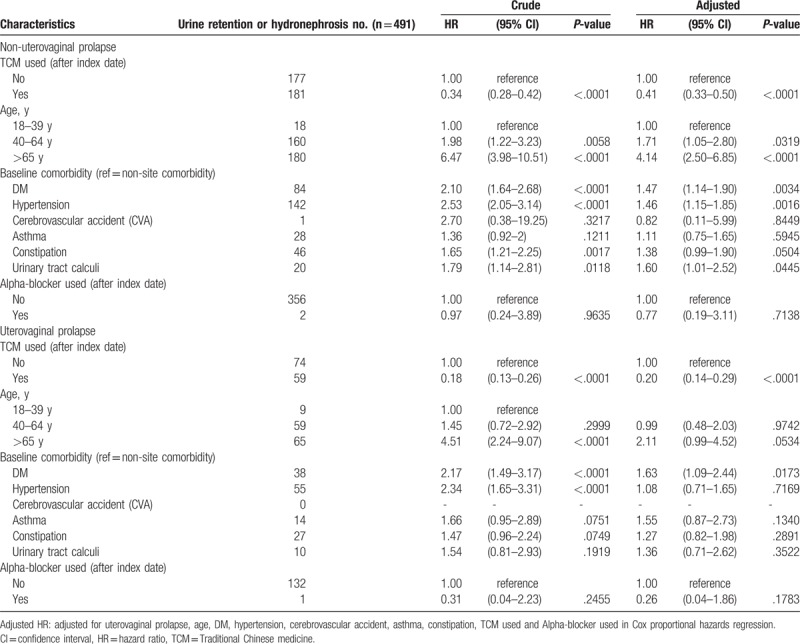
Cox model measured hazard ratio and 95% confidence intervals of urine retention or hydronephrosis associated with TCM used stratified by stage of uterovaginal prolapse.

**Figure 1:**
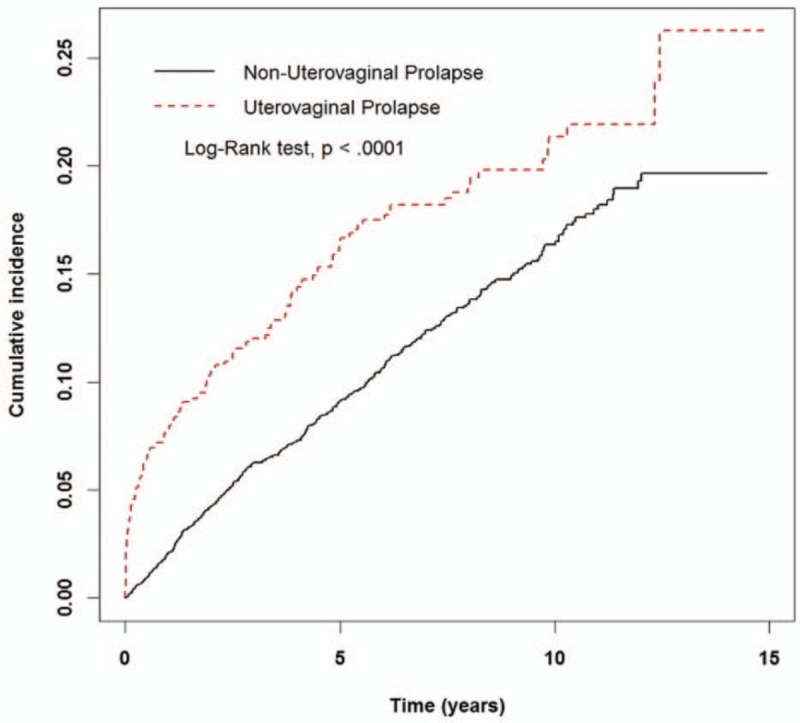
The estimated cumulative incidence of urine retention or hydronephrosis between the cohort and compared cohort group by Kaplan–Meier analysis.

**Figure 2:**
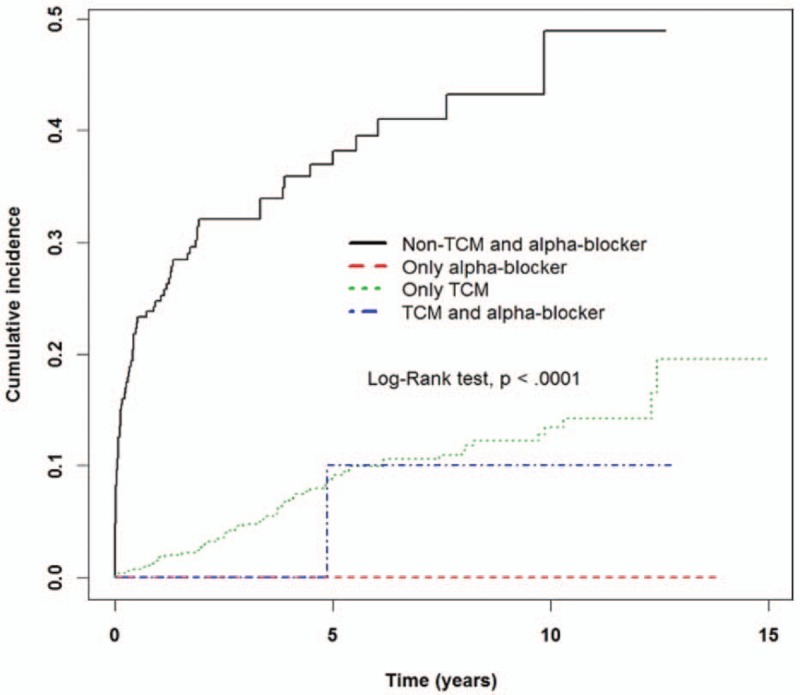
The estimated cumulative incidence of urine retention or hydronephrosis between those treated with TCM or alpha-blocker in the patients with uterovaginal prolapse cohort by Kaplan–Meier analysis.

The Kaplan–Meier analysis showed a higher incidence rate of urine retention or hydronephrosis in the uterovaginal prolapse cohort compared with that of the without uterovaginal prolapse cohort (Log rank test, *P* < .001) (Fig. 1). In the uterovaginal prolapse cohort, patients treated with only TCM showed a lower incidence rate of urine retention or hydronephrosis compared with whom treated with non-TCM and alpha-blocker (Fig. 2).

## 4 Discussion

Uterovaginal prolapse is known to be a cause of obstructive uropathy that can result in urine retention or hydronephrosis. According to previous studies, urinary splinting was reported by 5% to 12% of women with stage II anterior prolapse and 23% to 36% of those with stage III or IV anterior prolapse.^[8]^ The prevalence of hydronephrosis in women with advanced POP was 10.3% to 30.6%.^[32–34]^ Consistent with our findings that uterovaginal prolapse cohort has higher incidence rate of urine retention or hydronephrosis compared with cohort without uterovaginal prolapse (HR: 1.74, 95% CI: 1.43–2.14).

Chronic urine retention has the potential to cause renal damage if accompanied by high-pressure storage through the transmission of elevated bladder pressures to the upper urinary tract. Hydronephrosis may lead to renal dysfunction and even progress to irreversible renal damage.^[35]^ Hence, chronic urine retention or hydronephrosis from chronic ureteral kinking are indications for uterovaginal prolapse treatment.^[9]^ To our knowledge, this is the first study using a nationwide database to investigate the relationship between TCM use and obstructive uropathy in patients with uterovaginal prolapse. In the present study, we found that uterovaginal prolapse group using TCM had lower risk of urine retention or hydronephrosis than non-TCM users (HR: 0.2, 95% CI: 0.14–0.29). Compared with the other treatments, the use of alpha-adrenergic blocker medications was associated with relief of urinary retention in about 67% to 84% patients, using TCM had similar effect but no side effects including dizziness and rhinitis. The use of pessary was associated with relief of urinary retention in 75% patients, using TCM had similar effect but do not need to remove and clean any device. Pelvic reconstructive surgery was associated with relief of urinary retention in >90% patients, using TCM had less effect but without risk of complications and recurrence. The present study provides valuable evidence for medical benefits of TCM, and has implications for health policy.

In the present study, uterovaginal prolapse cohort has higher incidence of constipation. Chronic constipation is a risk factor for pelvic organ prolapse, probably due to repetitive increases in intraabdominal pressure.^[36]^ In the present study, uterovaginal prolapse cohort has higher incidence of urinary tract calculi. Long-standing pelvic organ prolapse caused urethral kinking, resulting in incomplete emptying of bladder leading to the urinary retention and providing the nidus and infectious environment required for stone development.^[37,38]^ POP is known to be a cause of obstructive uropathy that can result in urinary tract calculi. If urinary tract calculi obstruct at or distal to the renal pelvis, or at ureter may cause hydronephrosis.

In the present study, obstructive uropathy was significantly associated with a diagnosis of diabetes mellitus (HR: 1.52, 95% CI: 1.23–1.89), similar to previous studies’ findings that diabetes mellitus (DM) is clearly seen in a high percentage of women with obstructive uropathy.^[32,34]^ DM patients may have peripheral neuropathy complications and subsequently impairs bladder function, hence cause urine retention.^[39]^ Furthermore, as diabetes mellitus is associated with glomerulosclerosis and microvascular renal changes, these patients may be at increased risk for complications from the obstructive uropathy caused by POP.^[32]^

In the present study, obstructive uropathy was significantly associated with a diagnosis of hypertension (HR: 1.38, 95% CI: 1.13–1.7). One reason was that patients with obstructive uropathy may be present with hypertension. The mechanisms of elevated blood pressure may be that acute unilateral obstruction can cause hypertension via activation of the renin-angiotensin system whereas bilateral obstruction may elevate blood pressure through volume expansion.^[40,41]^ Another reason was that half the number of patients with obstructive uropathy were older than 65 years; the incidence of hypertension is associated with advancing age.

There are some limitations of the present study. First, many important information from uterovaginal prolapse patients such as serum biochemical data regarding stone disease, diet habits, cigarette or alcohol consumption, body weight, and family history of systemic disease was not disclosed in the NHIRD. Also, image data, including x-rays, urodynamic evaluation, and ultrasound, are not available in the NHIRD database, despite that these techniques are valuable techniques for investigating the progression of obstructive uropathy of uterovaginal prolapse. Second, the severity and disease duration of uterovaginal prolapse could not be evaluated simply by using the ICD-9-CM coding. Third, the relationship between TCM and obstructive uropathy may have been underestimated because the NHIRD database only included Chinese herbal medicine and acupuncture therapy prescriptions by licensed physicians. Other alternative Chinese medicines, such as natural and folk medicines or exercise therapy including Tai-Qi were not included in this study, and we were unable to incorporate this information in our study. Finally, the retrospective data generally has more confounding variables than the prospective clinical trials, further prospective studies are warranted to evaluate the relationship between TCM use and obstructive uropathy in uterovaginal prolapse patients.

In conclusions, the results of this nationwide population-based study support a relationship between TCM and a reduced risk of obstructive uropathy in uterovaginal prolapse women.

## Author contributions

**Conceptualization:** Wen-Chi Chen, Yuan-Chih Su, Huey-Yi Chen.

**Data curation:** Jen-Huai Chiang, Yung-Hsiang Chen.

**Formal analysis:** Jen-Huai Chiang.

**Funding acquisition:** Wen-Chi Chen, Yung-Hsiang Chen, Huey-Yi Chen.

**Investigation:** Yin-Jen Chang, Jen-Huai Chiang, Yuan-Chih Su, Kao-Sung Tsai, Ming-Yen Tsai, Yung-Hsiang Chen, Huey-Yi Chen.

**Methodology:** Huey-Yi Chen.

**Project administration:** Wen-Chi Chen, Huey-Yi Chen.

**Software:** Jen-Huai Chiang, Yuan-Chih Su.

**Supervision:** Wen-Chi Chen, Huey-Yi Chen.

**Validation:** Yin-Jen Chang, Jen-Huai Chiang, Kao-Sung Tsai, Kee-Ming Man, Ming-Yen Tsai, Yung-Hsiang Chen, Huey-Yi Chen.

**Writing – original draft:** Yin-Jen Chang.

**Writing – review & editing:** Wen-Chi Chen, Yung-Hsiang Chen, Huey-Yi Chen.

Yung-Hsiang Chen: 0000-0002-8756-5113.

Wen-Chi Chen: 0000-0002-7572-4201, Ming-Yen Tsai: 0000-0001-7722-3331.

## References

[1] Cormio L Mancini V Liuzzi G Surgical management of female pelvic organ prolapse with and without urinary incontinence: a single center experience. Medicine (Baltimore) 2017;96:e7914. 28953613 10.1097/MD.0000000000007914 PMC5626256

[2] Sun Y Zhang W Qie S Comprehensive comparing percutaneous endoscopic lumbar discectomy with posterior lumbar internal fixation for treatment of adjacent segment lumbar disc prolapse with stable retrolisthesis: a retrospective case-control study. Medicine (Baltimore) 2017;96:e7471. 28723757 10.1097/MD.0000000000007471 PMC5521897

[3] Handa VL Garrett E Hendrix S Progression and remission of pelvic organ prolapse: a longitudinal study of menopausal women. Am J Obstet Gynecol 2004;190:27–32. 14749630 10.1016/j.ajog.2003.07.017

[4] Hendrix SL Clark A Nygaard I Pelvic organ prolapse in the Women's Health Initiative: gravity and gravidity. Am J Obstet Gynecol 2002;186:1160–6. 12066091 10.1067/mob.2002.123819

[5] Nygaard I Bradley C Brandt D Pelvic organ prolapse in older women: prevalence and risk factors. Obstet Gynecol 2004;104:489–97. 15339758 10.1097/01.AOG.0000136100.10818.d8

[6] Bump RC Mattiasson A Bo K The standardization of terminology of female pelvic organ prolapse and pelvic floor dysfunction. Am J Obstet Gynecol 1996;175:10–7. 8694033 10.1016/s0002-9378(96)70243-0

[7] Lukacz ES DuHamel E Menefee SA Elevated postvoid residual in women with pelvic floor disorders: prevalence and associated risk factors. Int Urogynecol J Pelvic Floor Dysfunct 2007;18:397–400. 16804634 10.1007/s00192-006-0164-0

[8] Tan JS Lukacz ES Menefee SA Predictive value of prolapse symptoms: a large database study. Int Urogynecol J Pelvic Floor Dysfunct 2005;16:203–9. discussion 209. 15875236 10.1007/s00192-004-1243-8

[9] Jelovsek JE Maher C Barber MD Pelvic organ prolapse. Lancet 2007;369:1027–38. 17382829 10.1016/S0140-6736(07)60462-0

[10] Oksay T Ergun O Capar E Bilateral hydronephrosis secondary to cystocele. Ren Fail 2011;33:537–9. 21446783 10.3109/0886022X.2011.569104

[11] Chuang FR Lee CH Chen CS Bilateral moderate hydroureteronephrosis due to uterine prolapse: two case reports and review of the literature. Ren Fail 2003;25:879–84. 14575296 10.1081/jdi-120024303

[12] Delaere K Moonen W Debruyne F Hydronephrosis caused by cystocele. Treatment by colpopexy to sacral promontory. Urology 1984;24:364–5. 6237479 10.1016/0090-4295(84)90213-9

[13] Floyd MS Jr Casey RG Bredin HC Procidentia: a reversible cause of hydronephrosis in an 80-year-old woman. Int Urogynecol J Pelvic Floor Dysfunct 2008;19:1179–81. 18330482 10.1007/s00192-008-0587-x

[14] Kessler TM Studer UE Burkhard FC The effect of terazosin on functional bladder outlet obstruction in women: a pilot study. J Urol 2006;176:1487–92. 16952666 10.1016/j.juro.2006.06.009

[15] Low BY Liong ML Yuen KH Terazosin therapy for patients with female lower urinary tract symptoms: a randomized, double-blind, placebo controlled trial. J Urol 2008;179:1461–9. 18295277 10.1016/j.juro.2007.11.060

[16] Lee KS Han DH Lee YS Efficacy and safety of tamsulosin for the treatment of non-neurogenic voiding dysfunction in females: a 8-week prospective study. J Korean Med Sci 2010;25:117–22. 20052356 10.3346/jkms.2010.25.1.117 PMC2800025

[17] Romanzi LJ Chaikin DC Blaivas JG The effect of genital prolapse on voiding. J Urol 1999;161:581–6. 9915453

[18] Lazarou G Scotti RJ Mikhail MS Pessary reduction and postoperative cure of retention in women with anterior vaginal wall prolapse. Int Urogynecol J Pelvic Floor Dysfunct 2004;15:175–8. 15167996 10.1007/s00192-004-1138-8

[19] Fitzgerald MP Kulkarni N Fenner D Postoperative resolution of urinary retention in patients with advanced pelvic organ prolapse. Am J Obstet Gynecol 2000;183:1361–3. discussion 1363-4. 11120497 10.1067/mob.2000.110956

[20] Culligan PJ Nonsurgical management of pelvic organ prolapse. Obstet Gynecol 2012;119:852–60. 22433350 10.1097/AOG.0b013e31824c0806

[21] Yagi H Nishio K Sato R Clinical efficacy and tolerability of Gosha-jinki-gan, a Japanese traditional herbal medicine, for nocturia. J Tradit Complement Med 2015;6:126–9. 26870690 10.1016/j.jtcme.2014.11.021 PMC4737965

[22] Lin SK Lin PH Hsu RJ Traditional Chinese medicine therapy reduces the catheter indwelling risk in dementia patients with difficult voiding symptoms. J Ethnopharmacol 2017;203:120–6. 28359848 10.1016/j.jep.2017.03.040

[23] Kajiwara M Mutaguchi K Clinical efficacy and tolerability of gosha-jinki-gan, Japanese traditional herbal medicine, in females with overactive bladder. Hinyokika Kiyo 2008;54:95–9. 18323165

[24] Zheng XF Tian JS Liu P Analysis of the restorative effect of Bu-zhong-yi-qi-tang in the spleen-qi deficiency rat model using (1)H-NMR-based metabonomics. J Ethnopharmacol 2014;151:912–20. 24333365 10.1016/j.jep.2013.12.001

[25] Lin PH Lin SK Hsu RJ The use and the prescription pattern of Traditional Chinese Medicine among urolithiasis patients in Taiwan: a population-based study. J Altern Complement Med 2016;22:88–95. 26359806 10.1089/acm.2015.0116

[26] Mi J Duan J Zhang J Evaluation of antiurolithic effect and the possible mechanisms of Desmodium styracifolium and Pyrrosiae petiolosa in rats. Urol Res 2012;40:151–61. 21822640 10.1007/s00240-011-0401-y

[27] Tsai CH Pan TC Lai MT Prophylaxis of experimentally induced calcium oxalate nephrolithiasis in rats by Zhulingtang, a traditional Chinese herbal formula. Urol Int 2009;82:464–71. 19506417 10.1159/000218539

[28] Liu QL Sato S Kishikawa T Effectiveness of a traditional Chinese medicine, Wulingsan, in suppressing the development of nephrocalcinosis induced by a high phosphorus diet in young rats. Med Electron Microsc 2001;34:103–14. 11685659 10.1007/s007950170004

[29] Tsai CH Chen YC Chen LD A traditional Chinese herbal antilithic formula, Wulingsan, effectively prevents the renal deposition of calcium oxalate crystal in ethylene glycol-fed rats. Urol Res 2008;36:17–24. 18040675 10.1007/s00240-007-0122-4

[30] Nakagawa T Tashiro I Fujimoto M Keishibukuryogan reduces renal injury in the early stage of renal failure in the remnant kidney model. Evid Based Complement Alternat Med 2011;2011:914249. 19633031 10.1093/ecam/nep089 PMC3137790

[31] Nakagawa T Goto H Hikiami H Protective effects of keishibukuryogan on the kidney of spontaneously diabetic WBN/Kob rats. J Ethnopharmacol 2007;110:311–7. 17123761 10.1016/j.jep.2006.09.043

[32] Dancz CE Walker D Thomas D Prevalence of hydronephrosis in women with advanced pelvic organ prolapse. Urology 2015;86:250–4. 26194290 10.1016/j.urology.2015.05.005 PMC8605899

[33] Wee WW Wong HF Lee LC Incidence of hydronephrosis in severe uterovaginal or vault prolapse. Singapore Med J 2013;54:160–2. 23546030 10.11622/smedj.2013048

[34] Hui SY Chan SC Lam SY A prospective study on the prevalence of hydronephrosis in women with pelvic organ prolapse and their outcomes after treatment. Int Urogynecol J 2011;22:1529–34. 21822714 10.1007/s00192-011-1504-2

[35] Miyagi A Inaguma Y Tokoyoda T A case of renal dysfunction caused by pelvic organ prolapse. CEN Case Rep 2017;6:125–8. 28634833 10.1007/s13730-017-0257-2 PMC5694395

[36] Weber AM Walters MD Ballard LA Posterior vaginal prolapse and bowel function. Am J Obstet Gynecol 1998;179:1446–9. discussion 1449-50. 9855579 10.1016/s0002-9378(98)70008-0

[37] Dahiya P Gupta A Sangwan K Multiple bladder calculi: a rare cause of irreducible uterine prolapse. Arch Gynecol Obstet 2007;275:411–2. 17103181 10.1007/s00404-006-0272-6

[38] Wai CY Margulis V Baugh BR Multiple vesical calculi and complete vaginal vault prolapse. Am J Obstet Gynecol 2003;189:884–5. 14526336 10.1067/s0002-9378(03)00131-5

[39] Kaplan SA Te AE Blaivas JG Urodynamic findings in patients with diabetic cystopathy. J Urol 1995;153:342–4. 7815578 10.1097/00005392-199502000-00013

[40] Weidmann P Beretta-Piccoli C Hirsch D Curable hypertension with unilateral hydronephrosis. Studies on the role of circulating renin. Ann Intern Med 1977;87:437–40. 907242 10.7326/0003-4819-87-4-437

[41] Yeung SCJ, Escalante CP. Oncologic Emergencies: BC Decker, 2002.

